# Accelerated Evolution of Limb-Related Gene *Hoxd11* in the Common Ancestor of Cetaceans and Ruminants (Cetruminantia)

**DOI:** 10.1534/g3.119.400512

**Published:** 2019-12-02

**Authors:** Jun Li, Songyang Shang, Na Fang, Yubo Zhu, Junpeng Zhang, David M. Irwin, Shuyi Zhang, Zhe Wang

**Affiliations:** * Department of Obstetrics and Gynecology, Shengjing Hospital of China Medical University, Shenyang 110004, China,; † College of Animal Science and Veterinary Medicine, Shenyang Agricultural University, Shenyang 110866, China,; ‡ State Key Laboratory of Estuarine and Coastal Research, Institute of Estuarine and Coastal Research, East China Normal University, Shanghai 200062, China, and; § Department of Laboratory Medicine and Pathobiology, University of Toronto, Toronto, ON M5S 1A8, Canada

**Keywords:** Limb, carpal, tarsal, *5′Hox* genes, *Hoxd11*, mutation

## Abstract

Reduced numbers of carpal and tarsal bones (wrist and ankle joints) are extensively observed in the clade of Cetacea and Ruminantia (Cetruminantia). *Homebox D11* (*Hoxd11*) is one of the important genes required for limb development in mammals. Mutations in *Hoxd11* can lead to defects in particular bones of limbs, including carpus and tarsus. To test whether evolutionary changes in *Hoxd11* underlie the loss of these bones in Cetruminantia, we sequenced and analyzed *Hoxd11* coding sequences and compared them with other 5′ *HoxA* and *HoxD* genes in a taxonomic coverage of Cetacea, Ruminantia and other mammalian relatives. Statistical tests on the *Hoxd11* sequences found an accelerated evolution in the common ancestor of cetaceans and ruminants, which coincided with the reduction of carpal and tarsal bones in this clade. Five amino acid substitutions (G222S, G227A, G229S, A240T and G261V) and one amino acid deletion (G254Del) occurred in this lineage. In contrast, other 5′ *HoxA* and *HoxD* genes do not show this same evolutionary pattern, but instead display a highly conserved pattern of evolution in this lineage. Accelerated evolution of *Hoxd11*, but not other 5′ *HoxA* and *HoxD* genes, is probably related to the reduction of the carpal and tarsal bones in Cetruminantia. Moreover, we found two amino acid substitutions (G110S and D223N) in *Hoxd11* that are unique to the lineage of Cetacea, which coincided with hindlimb loss in the common ancestor of cetaceans. Our results give molecular evidence of *Hoxd11* adaptive evolution in cetaceans and ruminants, which could be correlated with limb morphological adaptation.

The numbers of carpal and tarsal bones in limbs have evolved differently among species (Hinchliffe and Johnson 1980) (Figure 1). The carpus of mammalian species normally have eight bones, consisting of a proximal row that includes the scaphoid, lunate, triquetral, and pisiform bones and a distal row that contains the trapezium, trapezoid, capitate, and hamate (Yalden 1971; Sadan and Misk 2014; Netter and Colacino 1989). The centrale is located between the two rows; However, this bone is fused with the trapezoid or with the scaphoid and lunate in some mammalian species (Kivell and Begun 2007; Stafford and Thorington 1998). The tarsus of mammals usually has seven bones, with a proximal row of the calcaneus and the talus and a distal row of a cuboid and three cuneiform bones, with a navicular bone between the two rows (Lewis 1980).

**Figure 1:**
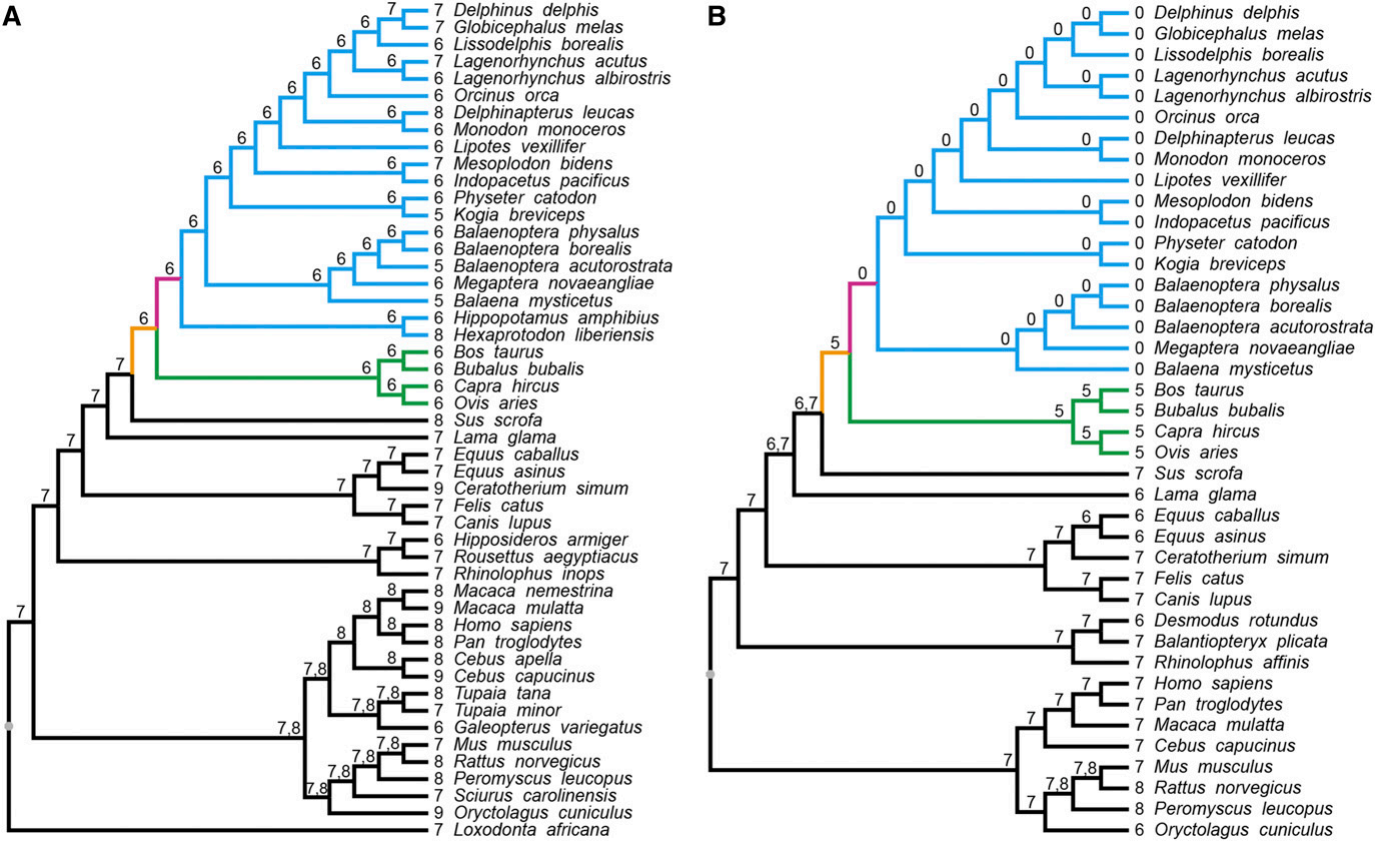
Evolution of carpal and tarsal bone numbers in mammals. (A) Carpus. (B) Tarsus. The numbers of carpal and tarsal bones in extant species are shown to the left side of the names of each species. Numbers for the ancestors are shown on the branches of the species tree. The lineage leading to Cetruminantia is shown in orange and the lineage leading to Cetacea is in pink. Clades for cetaceans and ruminants are indicated in blue and green, respectively.

The Cetruminantia belong to the order Cetartiodactyla and consists of diverse species, including cetaceans, hippopotamuses and ruminants. In species of this clade, the carpus normally has six bones due to the loss of the trapezium, although not in specific toothed whales, and the fusion of the trapezoid and magnum, with the exception of certain whale and hippopotamus (Cooper *et al.* 2007; Sadan and Misk 2014; Schellhorn and Pfretzschner 2014; Fisher *et al.* 2007; Turner 1909; Raven 1942; Turner 1885; Benham 1902). However, in the pig, which is a close relative of the Cetruminantia, the carpus has eight bones (Karan 2012). In the orders Perissodactyla and Carnivora, which are close to Cetartiodactyla, the carpus normally consists of seven bones with the rhinoceros even having nine carpal bones (Sadan and Misk 2014; Tung *et al.* 2010). For hindlimbs, ruminants normally have five tarsal bones, with the fusion of cuneiform bones and also cuboid and navicular (Rajtová 1974; Ehlert *et al.* 2011). Cetaceans even lost their hindlimbs to adapt to the aquatic lifestyle (Bejder and Hall 2002). In contrast to ruminants, the pig still has seven tarsal bones (Akers and Denbow 2008). Species in orders Perissodactyla and Carnivora have either six or seven tarsal bones (Alam El Din *et al.* 1986; Galateanu *et al.* 2014; Akers and Denbow 2008) (Figure 1).

Homeobox (*Hox*) genes encode transcription factors that control developmental patterning in animals (Pearson *et al.* 2005; He *et al.* 2018). In mammals, 39 *Hox* genes have been identified, which belong to the *HoxA*, *HoxB*, *HoxC* and *HoxD* clusters (Mallo 2018). Among these genes, some within the *HoxA* and *HoxD* clusters are especially critical for early limb patterning (Young *et al.* 2019; Moreau *et al.* 2019). Deletion of these two clusters causes severe limb truncation during development (Kmita *et al.* 2005). The *5′Hox* genes (groups 9-13) from these two clusters regulate limb patterning. Paralogous genes of each cluster are expressed in a sequential pattern during development (Zákány and Duboule 1999; Kmita *et al.* 2002). The *5′Hox* genes then participate in regulating the development of the most proximal stylopod, the middle zeugopod and the most distal autopod of limbs (Zákány and Duboule 2007).

Among the *Hox* genes, *Hoxd11* and *Hoxa11* are particularly important for distal limb development (Zákány and Duboule 1999). Expression of *Hoxa11* is mainly restricted to the zeugopod, while *Hoxd11* is expressed not only in the zeugopod, but also in the autopod (Koyama *et al.* 2010). *Hoxd11* is highly expressed at carpal, tarsal and digital regions of mammals during limb development (Wang *et al.* 2014; Booker *et al.* 2016). Mutations in *Hoxd11* can lead to abnormal ulna, radius, carpals, metacarpals and phalanges in forelimbs (Boulet and Capecchi 2004; Fleischman *et al.* 2002; Boulet and Capecchi 2002). More interestingly, although no obvious morphological changes happen in hindlimbs, *Hoxd11* does affect part of the tarsal bones (Davis and Capecchi 1994). Similarly, mutants of *Hoxa11* cause malformations of both forelimbs, as well as the ulna, radius and carpal bones, and tibia and fibula of the hindlimbs (Small and Potter 1993). Double mutations of *Hoxa11* and *Hoxd11* together caused even more severe morphological malformations, including the disappearance of some tarsal bones (Davis *et al.* 1995).

Thus, the *Hoxd11* gene is of particular interest in studies of the evolution of the mammalian carpal and tarsal bones. The evolution of the *Hoxd12* and *Hoxd13* genes, which are more related to the development of digits (Davis and Capecchi 1996), has been studied in better detail (Wang *et al.* 2009). Molecular evolutionary studies on mammalian *Hoxd12* and *Hoxd13* genes has been reported that the genes were associated with changes in digit and phalange numbers in cetaceans (Wang *et al.* 2009). Recent evolutionary studies provided further evidences that different *Hox* genes are important for the diversified morphologies during mammalian evolution (Liang *et al.* 2013; Nery *et al.* 2016; Li *et al.* 2018). Considering that *Hoxd11* is more specifically involved in carpal and tarsal bone development (Davis and Capecchi 1994; Koyama *et al.* 2010), we conducted a molecular evolutionary study of this gene to determine whether evolution of *Hoxd11* might explain the bone number variations in Cetruminantia.

## Materials and Methods

### Ethics approval

The collection procedure of mammalian samples in this study followed ethical principles and approved by the Animal Ethics Committee of Shenyang Agricultural University.

### Mammalian species coverage for Hoxd11

Tissue samples from 12 mammals, including 10 cetartiodactyls, were obtained. The samples cover eight cetacean species (Sowerby’s beaked whale, *Mesoplodon bidens*; Blainville’s beaked whale, *Mesoplodon densirostris*; Northern bottlenose whale, *Hyperoodon ampullatus*; Atlantic white-sided dolphin, *Lagenorhynchus acutus*; pygmy sperm whale, *Kogia breviceps*; humpback whale, *Megaptera novaeangliae*; fin whale, *Balaenoptera physalus*; common minke whale, *Balaenoptera acutorostrata*), two artiodactyls (goat, *Capra hircus*; pig, *Sus scrofa*), one perissodactyl (donkey, *Equus asinus*), and one bat (great leaf-nosed bat, *Hipposideros armiger*).

To trace the evolutionary history of *Hoxd11* in mammals, whole coding sequences for this gene were obtained from other 41 species representing orders Cetartiodactyla, Perissodactyla, Carnivora, Chiroptera, Primates, Rodentia, Lagomorpha and Afrosoricida. Whole coding regions of *Hoxd11* for these taxa were identified in GenBank (www.ncbi.nlm.nih.gov) using BLAST. The hippopotamus, the species in Camelidae and some other species were not included, because their *Hoxd11* sequences cannot be found in the genome data using the BLAST method or they were partial coding sequences.

Sequences from all 53 species were used to perform the evolutionary analyses, including comparisons of the highly variable region of *Hoxd11* and to reconstruct the ancestral states for amino acid sites. Detailed species information and GenBank accession numbers are listed in Supplemental Table S1.

### Hoxd11 gene amplification, cloning and sequencing

Genomic DNA was isolated from the 12 tissue samples using DNeasy Blood & Tissue Kits (Qiagen) and then used as template to amplify the *Hoxd11* coding regions. PCR were conducted for exon 1 and for most part of exon 2 separately. For exon 1, we designed one set of primers for 11 species (F1: 5′ ATG AAC GAC TTT GAC GAG TGC 3′ and R1: 5′ CCC TTC GAA CGC TTA TAA AGT A 3′) and another set of primers specifically for the great leaf-nosed bat (F2: 5′ GTG TGG GGA AT[G/C] GGA ACC TC 3′ and R2: 5′ CCA CCC ACC CGT AAA A[A/G]C AG 3′). For exon 2, we used one set of primers (F3: 5′ AAA GCG CTG TCC CTA CAC C 3′ and R3: 5′ CAG TGA CCC ATG CCT TGA TA 3′) for all 12 mammals. PCR products were purified using QIAquick Gel Extraction Kits (Qiagen), ligated into pGEM-T easy vectors (Promega) and then cloned using DH5α competent cells (Tiangen). Positive clones were sequenced on an ABI 3730 sequencer (Applied Biosystems).

### Species tree construction

We constructed a species tree for the analyses of the *Hox* genes, evolutionary history of bone numbers, and the focal amino acid sites according to previous studies. In detail, relationships of the orders was determined according to Springer *et al.* (Springer *et al.* 2003). For the relationships of the various species in each order, several references were used as none included all of the species used in this study. The Cetartiodactyla tree was constructed according to the following five references (McGowen *et al.* 2009; Hernández Fernández and Vrba 2005; Chen *et al.* 2019; Chen *et al.* 2018; O’Leary *et al.* 2013), the Perissodactyla tree was constructed according to Steiner and Ryder (Steiner and Ryder 2011), the Carnivora tree was constructed according to Nyakatura and Bininda-Emonds (Nyakatura and Bininda-Emonds 2012), the Chiroptera tree was constructed according to the following three references (Kawai *et al.* 2003; Teeling *et al.* 2005; Shi and Rabosky 2015), the Primates tree was constructed according to Perelman *et al.* (Perelman *et al.* 2011), the Rodentia tree was constructed according to the following three references (Romanenko *et al.* 2012; Fabre *et al.* 2017; Fabre *et al.* 2012), and the Afrotheria tree was constructed according to Kuntner *et al.* (Kuntner *et al.* 2011).

### Evolutionary analysis of the number of carpal and tarsal bones

Based on the published data for extant mammalian species (see details and references in the first two paragraphs of Introduction section), the evolutionary histories and the numbers of carpal and tarsal bones in the ancestral lineages were estimated using the parsimony method using Mesquite 2 (Maddison and Maddison 2001).

### Molecular evolution analyses on Hoxd11

Our new *Hoxd11* sequences and those from databases were aligned together using ClustalW in MEGA 5 software (Thompson *et al.* 1994; Tamura *et al.* 2011). To examine the possibility that *Hoxd11* experienced adaptive evolution in Cetruminantia, selective pressures acting on the coding sequences were estimated using the Codeml program in PAML 4 (Yang 2007). *ω* values (the nonsynonymous substitution rate [dN]/synonymous substitution rate [dS]) significantly greater than one represents positive selection; those equal to one are neutral evolution; and those less than one indicated purifying selection.

To test whether *Hoxd11* underwent natural selection in Cetruminantia, we carried out several “branch models” in Codeml. The one-ratio model assumes all branches have the same *ω* value, but the free-ratio model assumes variable *ω* values in different lineages (Yang 1998). The two-ratio model allows *ω* to have different (but same within each group) values in the foreground and background branches (Yang 1998). Foreground lineage tested was the ancestor leading to Cetruminantia (termed branch “C” in this study). Codeml was also used to infer ancestral sequences and amino acid substitutions.

To visualize variation in the *ω* values along the *Hoxd11* coding region, we undertook a sliding window analysis using SWAAP 1 (Pride 2000). Parameters for window size was set as 75 bp with a step size of 12 bp, and *ω* values were calculated by the Nei and Gojobori’s method (Nei and Gojobori 1986). Based on the 53 species dataset of *Hoxd11*, evolutionary histories of the focal amino acid sites in the ancestral lineage of Cetruminantia were estimated using the parsimony method in Mesquite 2 (Maddison and Maddison 2001).

### Molecular evolution of other 5′ HoxA and HoxD genes

The two-ratio and one-ratio models were also applied to other 5′ *HoxA* and *HoxD* genes to determine whether these genes underwent the same evolutionary pattern as *Hoxd11* in the ancestor of Cetruminantia. The coding sequences for the genes were retrieved from GenBank using BLAST with accession numbers shown in Supplemental Table S2. The datasets were aligned by MEGA 5 (Tamura *et al.* 2011; Thompson *et al.* 1994) and analyzed by PAML 4 as mentioned above (Yang 2007, 1998).

### Data availability

The newly sequenced *Hoxd11* used in this study are available in GenBank with accession numbers KU842353-KU842364. We have uploaded supplementary material to figshare. File S1 contains species trees for the evolutionary analyses of other 5′ *HoxA* and *HoxD* genes (Figure S1) and GenBank accession numbers of *Hoxd11* sequences from 53 mammalian species analyzed in this study (Table S1). File S2 contains GenBank accession numbers of other 5′ *HoxA* and *HoxD* genes analyzed in this study (Table S2). Supplemental material available at figshare: https://doi.org/10.25387/g3.11278271.

## Results

### Variation in carpus and tarsus bone number in mammals

Although the numbers of carpal and tarsal bones vary in extant mammalian species, they only changed once (carpus) or twice to four times (tarsus) from remote ancestors. The numbers of bones in both the carpus and tarsus has been reduced by at least one bone in the common ancestor of cetaceans and ruminants (Figure 1). In the common ancestor of all modern cetaceans, the numbers of tarsal bones was reduced from five to zero (Figure 1B).

### Mammalian Hoxd11 gene sequences

We successfully cloned and sequenced ∼93% of the protein coding region of *Hoxd11* from ten Cetartiodactyla and two other mammalian species. Taken together with whole coding sequences downloaded from public databases, a total of 53 mammalian *Hoxd11* sequences, including sequences from 18 species of Cetruminantia, were analyzed in this study. The full set of sequences from 53 species was used for the PAML analyses, reconstruction of ancestral states for amino acid sites in Cetruminantia, and an alignment of the highly variable region of *Hoxd11*. Detailed taxonomic and gene information are listed in Supplemental Table S1.

### Molecular evolution of mammalian Hoxd11

**Figure 2:**
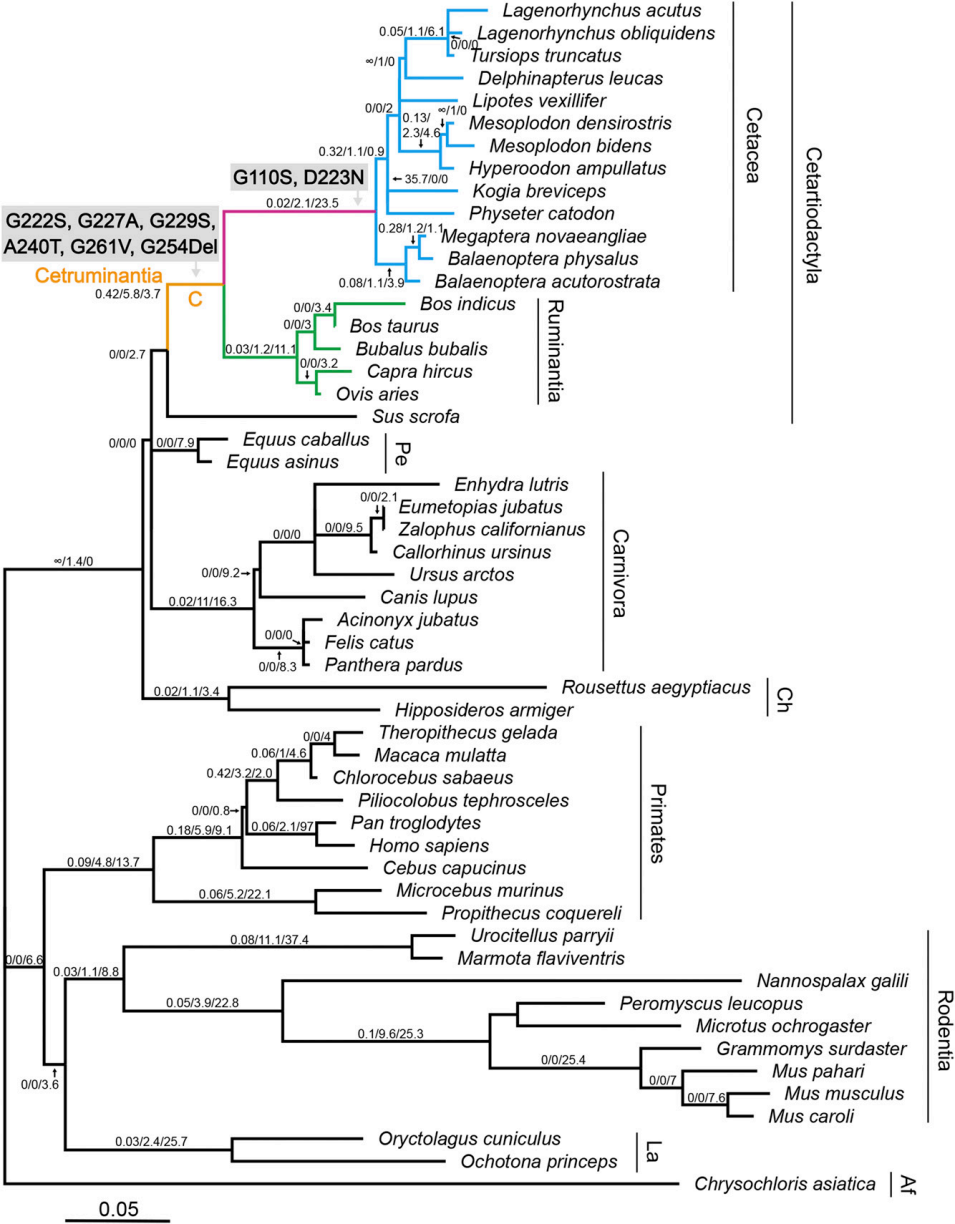
Molecular evolution of *Hoxd11* in mammalian ancestors. Branch lengths are proportional to nucleotide substitutions per codon. The *ω* values, numbers of nonsynonymous and synonymous substitutions inferred from the free-ratio model are shown on the species tree (the three numbers on the branches). The five amino acid substitutions on the lineage leading to Cetruminantia (branch “C”), shown in orange, and the two amino acid substitutions on the lineage leading to Cetacea, shown in pink, were mapped onto the branches. The clades for cetaceans and ruminants are indicated in blue and green respectively. Abbreviations for taxonomy are “Pe” (Perissodactyla), “Ca” (Carnivora), “Ch” (Chiroptera), “La” (Lagomorpha) and “Af” (Afrosoricida).

**Figure 3:**
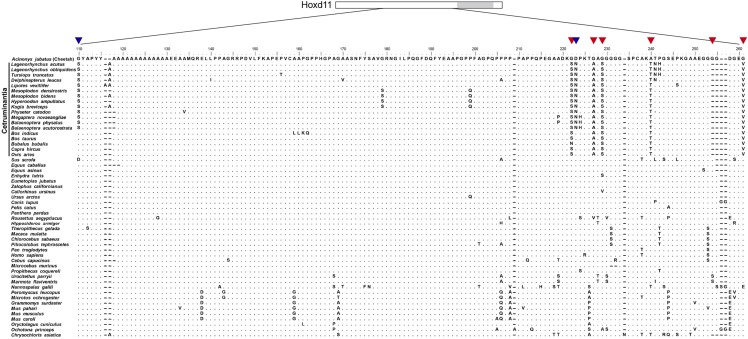
Alignment of mammalian Hoxd11 protein sequences. Partial amino acid sequences from 53 mammalian species are aligned. The five amino acid substitutions and one amino acid deletion that occurred in the ancestor of Cetruminantia are highlighted by the red arrows. The two amino acid substitutions that occurred in the ancestor of Cetacea are highlighted by the blue arrows. The gray box in the schematic of Hoxd11 is homeodomain.

**Figure 4:**
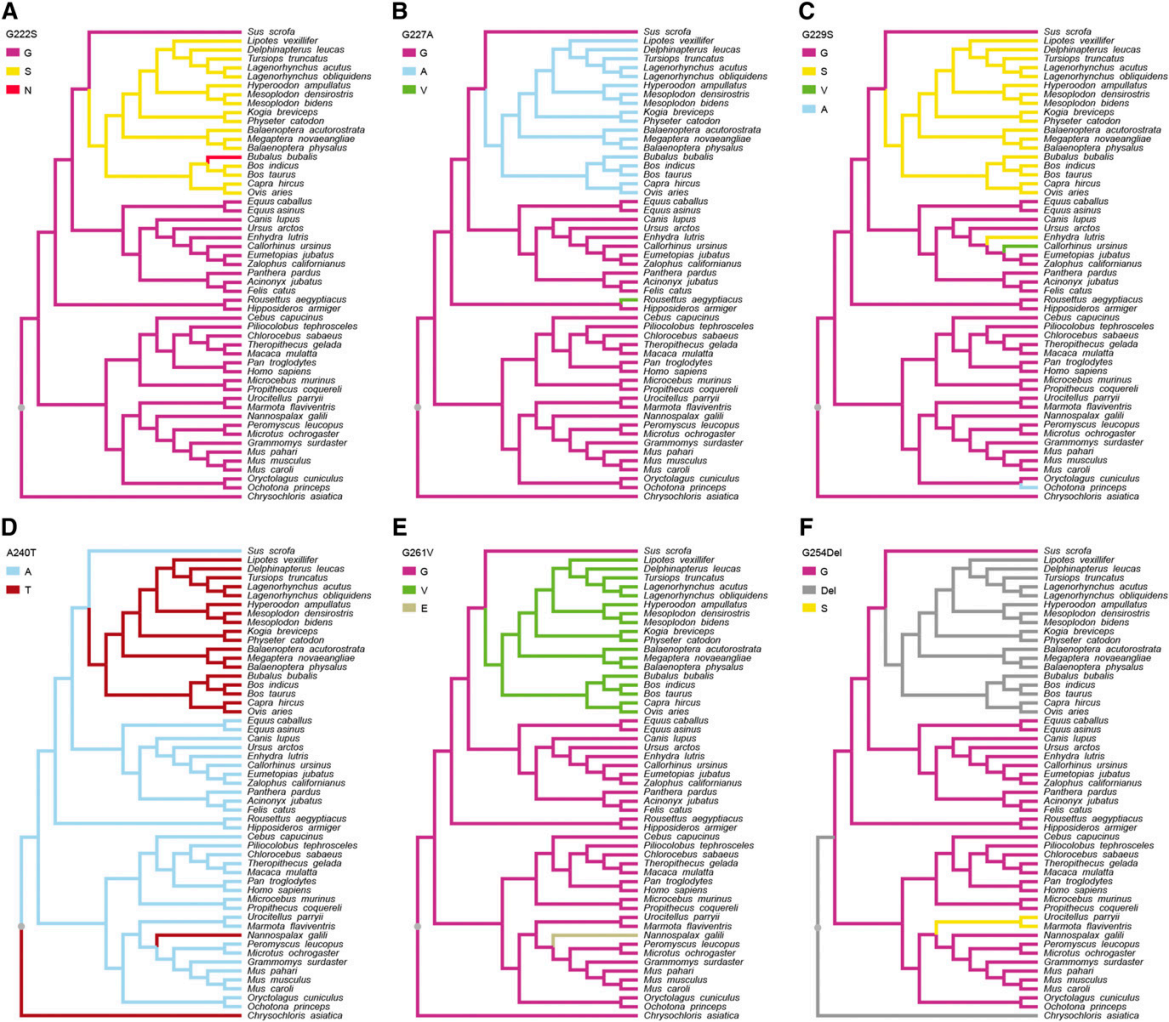
Evolution of the six amino acids in Hoxd11 proteins with mutations that occurred on the Cetruminantia branch. Evolutionary changes in the 53 mammals at the sites of the amino acid substitutions or deletion G222S, G227A, G229S, A240T, G261V and G254Del are shown in A, B, C, D, E and F, respectively.

**Figure 5:**
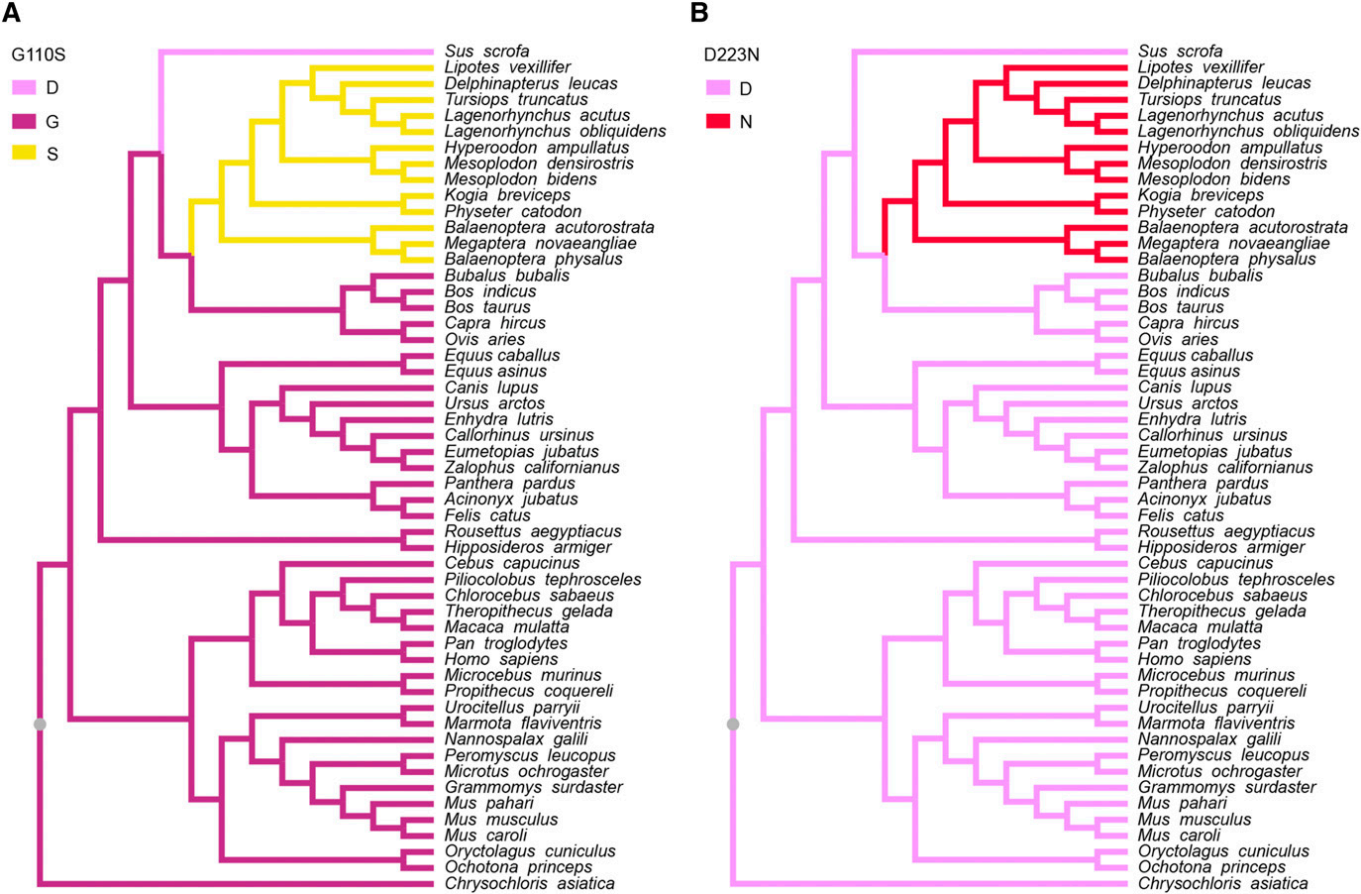
Evolution of the two amino acids that are substituted on the branch leading to Cetacea in the Hoxd11 protein. Evolutionary changes in the 53 mammals at the sites of the amino acid substitutions G110S and D223N are shown in A and B, respectively.

As a first step in examining the evolution of *Hoxd11* we assessed the levels of selection that had acted upon this sequence using PAML (Yang 2007). For the branch models, the free-ratio model was significantly better than the one-ratio model (*P* = 0.0008), indicating that selective pressures differed among the mammalian lineages. We identified five amino acid substitutions (G222S, G227A, G229S, A240T and G261V) and one amino acid deletion (G254Del), located adjacent to the conserved homeodomain, which occurred on the branch leading to Cetruminantia, and two amino acid substitutions (G110S and D223N) that occurred on the branch leading to Cetacea (Figures 2 and 3). To further investigate the evolution of these eight amino acid substitutions or deletions, we aligned the Hoxd11 protein sequences from the 53 species (Figure 3) and separately traced the evolutionary history of each substitution site (Figures 4 and 5). Of the eight amino acid substitutions, three (G222S, G227A and G261V) are exclusive to Cetruminantia, and two (G110S and D223N) are exclusive to Cetacea, while the remaining three occur not only in cetaceans and ruminants, but also in several other mammalian species.

Detailed *ω* values, nonsynonymous and synonymous substitution numbers inferred from the free-ratio model for each branch are shown in Figure 2. The branch leading to Cetruminantia shows the highest *ω* value (ω = 0.42) among the ancestral lineages that have more than 0 synonymous substitutions. Specifically, on this branch the number of nonsynonymous substitutions (dN = 5.8) is higher than on most ancestral lineages, and the number of synonymous substitutions (dS = 3.7) is much lower than on other ancestral lineages (dS = 9.1 to 37.4) that have higher numbers of nonsynonymous substitutions.

**Figure 6:**
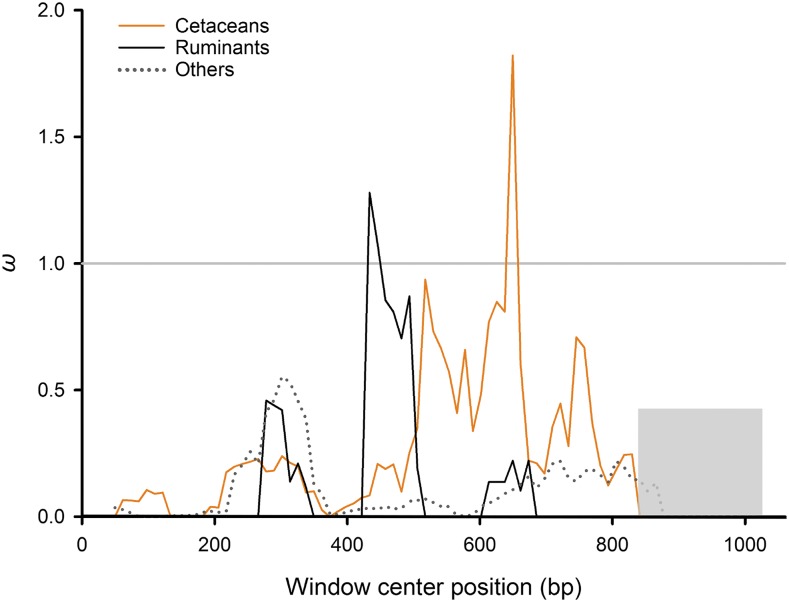
Sliding window analysis of *Hoxd11*. *ω* values were calculated in a sliding window across the *Hoxd11* sequence. The orange line represents *ω* values within cetaceans, the black line is ruminants, and the dotted gray line is other mammals. The homeobox is shown in the gray rectangle.

When the two-ratio test was applied, it revealed that the lineage leading to Cetruminantia has a greater than five times larger *ω* value (0.3839) than those for other branches (0.0712; *P* = 0.0215; Table 1). A sliding window analyses across the protein-coding region of *Hoxd11* shows that the *ω* value changes along the gene. This result showed that there is a highly variable region (*ω* > 1) in *Hoxd11* sequences of cetaceans and ruminants, but not in other mammals, which is adjacent to the conserved homeodomain (Figure 6).

**Table 1:** Branch model parameters for 5′ *HoxA* and *HoxD* genes

Gene	Model	*ω*_0_	*ω*_C_	2Δ*ℓ*	*P* value
*Hoxa9*	One-ratio (*ω*_0_)	0.0483	= *ω*_0_		
	Two-ratio (*ω*_0_, *ω*_C_)	0.0483	0.0993	0.3076	0.5791
*Hoxa10*	One-ratio (*ω*_0_)	0.0863	= *ω*_0_		
	Two-ratio (*ω*_0_, *ω*_C_)	0.0863	0.2286	0.4007	0.5267
*Hoxa11*	One-ratio (*ω*_0_)	0.0675	= *ω*_0_		
	Two-ratio (*ω*_0_, *ω*_C_)	0.0675	0.4016	0.0002	0.9885
*Hoxa13*	One-ratio (*ω*_0_)	0.0136	= *ω*_0_		
	Two-ratio (*ω*_0_, *ω*_C_)	0.0136	0.0001	0.3530	0.5524
*Hoxd9*	One-ratio (*ω*_0_)	0.1073	= *ω*_0_		
	Two-ratio (*ω*_0_, *ω*_C_)	0.1073	0.1558	0.0596	0.8071
*Hoxd10*	One-ratio (*ω*_0_)	0.1230	= *ω*_0_		
	Two-ratio (*ω*_0_, *ω*_C_)	0.1230	0.0001	0.6032	0.4374
***Hoxd11***	One-ratio (*ω*_0_)	0.0712	= *ω*_0_		
	**Two-ratio (*ω*_0_, *ω*_C_)**	**0.0712**	**0.3839**	5.28	0.0215*
*Hoxd12*	One-ratio (*ω*_0_)	0.1050	= *ω*_0_		
	Two-ratio (*ω*_0_, *ω*_C_)	0.1050	0.0001	1.0701	0.3009
*Hoxd13*	One-ratio (*ω*_0_)	0.0417	= *ω*_0_		
	Two-ratio (*ω*_0_, *ω*_C_)	0.0417	0.0001	0.7609	0.3830

*ω*_C_ is for the common ancestor of Cetruminantia in Figure 2 (the foreground branch). *ω*_0_ is for all other branches (the background branches). *, *P* value of *Hoxd11* is significant (<0.05). Significantly different *ω* values for *Hoxd11* are shown in bold.

### Molecular evolution of other 5′ HoxA and HoxD genes

For other 5′ *HoxA* and *HoxD* genes, complete coding sequences from representative species were analyzed. Two-ratio tests specifically on the ancestral branch leading to Cetruminantia indicate none of the genes shown an *ω* value significantly different from background branches (Table 1 and Supplemental Figure S1).

## Discussion

In mammals, reduced numbers of carpal and tarsal bones are extensively observed in species of Cetruminantia (Cooper *et al.* 2007; Benham 1902; Schellhorn and Pfretzschner 2014; Sadan and Misk 2014; Rajtová 1974; Ehlert *et al.* 2011). To uncover the genetic basis and evolution of the bone reductions, we reconstructed a phylogenetic tree of the phenotypic changes (Figure 1) and performed molecular evolutionary analyses focusing on the *Hoxd11* gene (Figure 2 and Table 1). The two-ratio model analysis shows that *Hoxd11* had a significantly higher *ω* value in the common ancestral lineage of Cetruminantia than those in other lineages. Five nonsynonymous substitutions (G222S, G227A, G229S, A240T and G261V; four of them are related to the loss of glycine) and one amino acid deletion (G254Del; loss of glycine) occurred on the ancestral branch for Cetruminantia. Two mutations (G110S and D223N) occurred on the ancestral branch for Cetacea that are unique to this clade. These eight mutations are located adjacent to the conserved homeodomain in *Hoxd11*, and three of them transform hydrophilic amino acids to hydrophobic residues (G227A and G261V) or vice versa (A240T). In addition, the sliding window analysis show dramatic differences between Cetruminantia and other mammals. As *Hoxd11* is a transcription factor that is typically composed of a DNA-binding domain and an activator domain, the area containing the mutations might correspond to the latter domain and thus influence the efficiency of DNA binding and thus the expression of its own gene and of downstream genes (Berg *et al.* 2002). These results indicate that the mutations might be due to positive selection and result in the bone losses or due to relaxed selective constraint. Given high expression of *Hoxd11* in mammalian limbs and functional importance of *5′HoxD* genes on limb development (Kmita *et al.* 2005; Gehrke *et al.* 2015; Fabre *et al.* 2018; Bickelmann *et al.* 2017; Booker *et al.* 2016), it is unlikely that *Hoxd11* underwent relaxed purifying selection during the origin of Cetruminantia, however, it possible that this occurred in the evolution of the ancestral cetaceans, as this is when hindlimb loss occurred. *Hoxd11* is required for positioning of the pelvic girdle in mice (Davis and Capecchi 1994). Reductions in the hindlimb and pelvic girdle development in Cetaceans may also reflect changes in the Hoxd11 amino acid sequence and expression patterns. The consequences of changes in gene expression cannot be directly investigated in developing embryos of Cetruminantia, however, deregulated premature expression of *Hoxd11-13* in mice can induce massive defects in the proximal limbs of mice, including the carpus (Zákány *et al.* 2007), indicating that relaxed selective constraint might be associated with the loss of the hind limbs in Cetacea.

In a previous study we reported that the *Hoxd12* and *Hoxd13* genes underlie the origin and diversification of the cetacean flipper (Wang *et al.* 2009). However, little consideration was paid to *Hoxd11* evolution and its role in the patterning of carpal and tarsal bones. In this study, we provided evidence on the adaptive evolution of *Hoxd11* and suggested a role for this gene in the reduction in the number of carpal and tarsal bones in Cetruminantia.

Mice with mutations in *Hoxd11* have been reported to possess fusions between specific carpal bones (Davis and Capecchi 1994; Favier *et al.* 1995). However, since the development of the limb involves the complex of *Hox* genes and their interactions, *Hoxd11* also likely works together with other genes to implement the reduction in the number of carpal and tarsal bones (Zákány and Duboule 2007). For example, the mice with double mutations in *Hoxa11* and *Hoxd11* can also have carpal bone fusions (Davis *et al.* 1995). Mutant mice involving both the *Hoxd11* and *Hoxd13* genes display a fusion of distal carpal bones (Davis and Capecchi 1996). In this study, the analyses on other 5′ *HoxA* and *HoxD* genes shown the genes are highly conservative in mammals, and none of them underwent accelerated evolution in the common ancestor of Cetruminantia. The results suggest that only *Hoxd11*, rather than other 5′ *HoxA* and *HoxD* genes, may underlay critical morphological modification during the evolution of Cetruminantia limb patterning.

In conclusion, the *Hoxd11* gene sequences exhibit a significant accelerated evolution during the origin of Cetruminantia. Moreover, other 5′ *HoxA* and *HoxD* genes show highly conserved evolution in this lineage, which supports a potentially important role of *Hoxd11* in Cetruminantia evolution. Accelerated evolution of *Hoxd11* in the ancestor of Cetruminantia was probably associated with the reduction of the carpal and tarsal bones in this lineage, whereas the mutations that are unique to the ancestor of Cetacea are probably related to Cetacean hindlimb loss. Further experimental analyses would be necessary to determine the exact function of the eight mutations observed in Cetruminantia.
